# Phenotype analysis of families with *TP53* germline variants at the Center for Familial Breast and Ovarian Cancer, Cologne

**DOI:** 10.1002/cam4.6920

**Published:** 2024-01-17

**Authors:** K. Kast, K. Rhiem, M. Larsen, B. Wappenschmidt, R. Schmutzler

**Affiliations:** ^1^ Center for Hereditary Breast and Ovarian Cancer, Center for Integrated Oncology (CIO), Medical Faculty University Hospital Cologne Cologne Germany

**Keywords:** breast cancer, cancer prevention, genetic variants, next‐generation sequencing

## Abstract

**Purpose:**

Tumor protein p53 *(TP53*) pathogenic variant (PV) carriers are identified during genetic testing for hereditary causes of cancer. PVs in *TP53* are associated with the Li‐Fraumeni syndrome (LFS), and thus, surveillance and preventive measures are important for *TP53* PV carriers. However, the penetrance of *TP53* PVs can be low if the Chompret criteria are not fulfilled. In this study, we compared the phenotypic characteristics of families that did and did not fulfill the LFS criteria according to Chompret.

**Methods:**

The German Consortium for Hereditary Breast and Ovarian Cancer (GC‐HBOC) database was used to identify index patients with a likely pathogenic/pathogenic *TP53* variant and their family members. The study investigated the type of variant, pedigree, age of onset, number of primary tumors, and histological type of BC.

**Results:**

*TP53* PV were present in the index cases of 35 families, 57% (20/35) of which fulfilled the Chompret criteria. The median age of onset at first BC diagnosis was lower in families that fulfilled the Chompret criteria compared to those who did not. Four of all diseased individuals were minors (4%; 4/105) when malignancy was first diagnosed. Sarcomas and brain tumors occurred in 10% (10/105) and in 7% (7/105) of all diseased persons, respectively. BC was the most frequently occurring first tumor (60%; 62/105) and additional malignancy (45%; 20/44) in this cohort. Subsequent malignancies developed in 31% (20/65) of the individuals who fulfilled the Chompret criteria compared with 15% (6/40) of those who did not.

**Conclusion:**

The tumor spectrum and age of onset found in this study showed that tumors other than BC had low disease penetrance in *TP53* PV carriers identified using the GC‐HBOC criteria for genetic testing.

## Graphical abstract

TP53‐associated tumor syndrome is a rare predisposition syndrome. Our analysis of the tumor spectrum and age of onset in 35 index patients supports the evidence for a less severe phenotype in families identified by gene panel testing for hereditary breast and ovarian cancer.

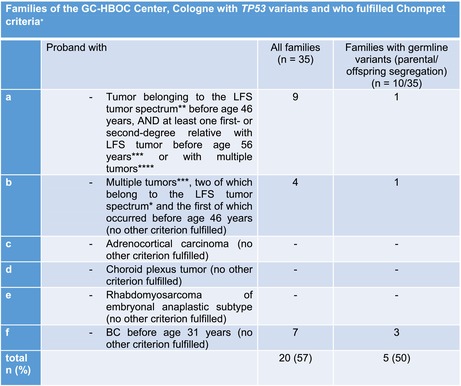

## 1 INTRODUCTION

Pathogenic variants (PV) of the *tumor protein p53* (*TP53*) gene are associated with the Li‐Fraumeni syndrome (LFS), which is currently gaining clinical attention even though it occurs by far less frequently than the Hereditary Breast and Ovarian Cancer (HBOC) syndrome (Li 1988, Ford 1998). The phenotypic manifestations of LFS include sarcomas, brain tumors, and other malignancies that occur both in childhood and adulthood.^1^, ^2^, ^3^

Premenopausal breast cancer (BC) is the most commonly occurring malignant tumor in female carriers of pathogenic *TP53* germline variants.^1^ It is common for patients with LFS to have multiple tumors, and increased radiation sensitivity with secondary malignancies that occur within the former radiation field is a unique feature of the syndrome.^4^ Additionally, it has been reported that patients with LFS are at an elevated risk of chemotherapy‐induced leukemia.^5^ Therefore, it is recommended that BC patients with pathogenic *TP53* germline variants avoid radiotherapy and genotoxic cytostatic drugs such as carboplatin.^5^, ^6^, ^7^ International guidelines recommend that an extensive multimodal screening program be applied for the prevention of secondary tumors in such patients.^8^, ^9^, ^10^ The “Toronto protocol” provides different examinations for children and adults, the application of which has resulted in improved overall patient survival.^11^, ^12^ However, the program is extensive and involves frequent examinations, which places a considerable psychological burden on the patients.^13^

In LFS, the lifetime probability of disease is reported to be almost 100% for both males and females.^3^ The term “hereditary *TP53*‐associated tumor syndrome” was suggested by the European Reference Network (ERN) GENTURIS to account for growing evidence of the variable penetrance observed in *TP53* germline carriers.^9^, ^14^, ^15^ Modern sequencing techniques can be used to identify germline *TP53* variant carriers in different diagnostic settings. Recent studies indicated the presence of a less severe phenotype in families that do not fulfill the adapted Chompret criteria for LFS (Table 1).^15^, ^16^ Moreover, recent analysis of the population prevalence of *TP53* variant carriers among patients without cancer in a gnomAD dataset suggested lower penetrance than previously predicted.^17^

**TABLE 1:** Number of families who fulfilled the adapted Chompret criteria for LFS and family constellation of *TP53* variant carriers.

Families of the GC‐HBOC Center, Cologne with *TP53* variants and who fulfilled Chompret criteria^a^			
	Proband with	All families (*n* = 35)	Families with germline variants (parental/offspring segregation) (*n* = 10/35)
a	Tumor belonging to the LFS tumor spectrum^b^ before age 46 years, AND at least one first‐ or second‐degree relative with LFS tumor before age 56 years^c^ or with multiple tumors^d^	9	1
b	Multiple tumors^c^, two of which belong to the LFS tumor spectrum^a^ and the first of which occurred before age 46 years (no other criterion fulfilled)	4	1
c	Adrenocortical carcinoma (no other criterion fulfilled)	–	–
d	Choroid plexus tumor (no other criterion fulfilled)	–	–
e	Rhabdomyosarcoma of embryonal anaplastic subtype (no other criterion fulfilled)	–	‐
f	BC before age 31 years (no other criterion fulfilled)	7	3
Total *n* (%)		20 (57)	5 (50)

Abbreviations: GC‐HBOC, German Consortium for Hereditary Breast and Ovarian Cancer, LFS, Li‐Fraumeni syndrome.

^a^ According to Bougeard et al.^1^

^b^ Premenopausal breast cancer (BC), soft tissue sarcoma, osteosarcoma, CNS tumor, adrenocortical carcinoma, leukemia, lung adenocarcinoma.

^c^ Except BC, if the proband has BC.

^d^ Except multiple BC.

With the introduction of multigene panel analysis at the German Consortium for Hereditary Breast and Ovarian Cancer (GC‐HBOC), the *TP53* gene has been routinely investigated in families that meet the criteria for germline testing (Table 2). These criteria are part of the German guidelines for BC screening, diagnosis, treatment, and follow‐up.^18^, ^19^ In an earlier study, we reported the prevalence of pathogenic *TP53* germline variants in 0.2%–0.3% of these families.^20^ This retrospective study aimed at investigating PV carrier phenotypes and genotypes by analyzing familial constellations, frequency and age of onset of malignancies, and variant types in families with *TP53* variants at the GC‐HBOC Center, Cologne.

**TABLE 2:** Family constellations of *TP53* variant carriers who fulfilled different GC‐HBOC criteria and the adapted Chompret criteria.

GC‐HBOC gene panel analysis criteria^a^	Families with GC‐HBOC‐criteria fulfilled		
	One or more GC‐HBOC‐criteria fulfilled, *n* = 35 (%)	One or more adapted Chompret criteria fulfilled^b^	
		Yes, *n* = 20 (57%)	No, *n* = 15 (43%)
3= > BC	10 (29)	5 (25)	5 (33)
2x BC, with 1x < 51 y	24 (69)	15 (75)	9 (60)
BC + OC	2 (6)	2 (10)	0
2x OC	0	0	0
mBC + BC	0	0	0
mBC + OC	0	0	0
BC < 36 y	20 (57)	16 (80)	4 (27)
bBC < 51 y	9 (26)	6 (30)	3 (20)
BC/OC	1 (3)	1 (5)	0
TNBC < 50 y^c^	5 (14)	3 (15)	2 (13)
OC (epithelial) < 80 y^c^	1 (3)	3 (15)	0
Total	72 (206)	51 (255)	23 (153)

^a^ According to Wöckel et al., 2018,^19^; Kast et al., (2016).^18^

^b^ See Table 1.

^c^ GC‐HBOC criterion since 01/2019.

Abbreviations: BC, breast cancer; bBC, bilateral breast cancer; GC‐HBOC, German Consortium for Hereditary Breast and Ovarian Cancer; mBC, male breast cancer TNBC, triple negative BC; OC, ovarian cancer; y, age of onset in years.

## 2 METHODS

Starting in July 2020, families associated with the GC‐HBOC Center of Cologne that have a documented pathogenic/likely pathogenic *TP53* variant (class 4/5) were identified using the GC‐HBOC database. Genetic testing results were matched with the clinical data available at the center.

### 2.1 Genetic analysis

Molecular genetic analysis was performed using DNA extracted from blood lymphocytes derived from index patients with breast and/or ovarian cancer according to the GC‐HBOC standard.^18^, ^19^, ^20^ In cases of affected family members who had died, normal tissue or tumor tissue embedded in paraffin was used for DNA extraction, if available. Next‐generation sequencing analysis of the breast and ovarian cancer genes, including *ATM*, *BARD1*, *BRCA1*, *BRCA2*, *CDH1*, *CHEK2*, *PALB2*, *RAD51C*, *RAD51D*, and *TP53*, was performed using the TruRisk® panel. Three patients had previously known *TP53* variants that were detected using gene panel analysis by an external laboratory.

### 2.2 Exclusion of families

*TP53* variants were rereviewed, and their pathogenicity was classified (class 1–5 according to^21^) using the current ACMG/AMP guidelines for *TP53* variants and the expert panel for variants of unknown significance (VUS task force) provided by the GC‐HBOC (Table S1).^21^, ^22^, ^23^, ^24^ Two patients were excluded from the study because they had class 3 variants. Patients suspected to have mosaicism or clonal hematopoiesis were also excluded^25^: Two patients with a variant fraction (VF) lower than 30% and suspected mosaicism were excluded, and four patients were excluded because of clonal hematopoiesis.^25^, ^26^, ^27^, ^28^ Another two patients were excluded because of unclear results with suspected somatic variants in tumor tissues.

### 2.3 Detection of malignancies

All tumors from the index patients and their family members from both family branches were recorded by the ICD‐10 code. If a metachronic ipsilateral second BC or ductal cancer in situ (DCIS) was diagnosed, a new primary was assumed in the case of the change of the histologic subtype (e.g., no special type vs. invasive lobular) or if the second event appeared after an interval of ≥10 years. In addition to the age at disease onset, histologic subtype and grading were recorded for BC. When available, histopathologic data also included hormone and HER2neu receptor status. Information about chemotherapy received by the patients, including the applied cytostatic drugs and the time interval between the start of chemotherapy and drawing of the blood sample for genetic analysis were also recorded.

### 2.4 Determination of the segregating family branch

For a given family, the family branch where the *TP53* variant was presumably segregating was selected based on the occurrence of core LFS cancers, including soft tissue and bone sarcoma, brain tumor, BC, adrenocortical carcinoma, leukemia, and tumor onset before the age of 50 years. If genetic testing of both parents yielded negative results, only the offspring were included as family members. In these families with assumed de novo *TP53* PVs, paternity was not examined further.

### 2.5 Clinical data

Clinical data of the index patients included surgical therapy for breast tumors and distinguished between breast‐conserving therapy and mastectomy and contralateral risk‐reducing mastectomy. All index patients were unrelated, and the family members of each patient were mutually exclusive.

### 2.6 Variant data

PVs were defined as missense variants (e.g., dominant negative, low‐penetrance, other missense) or loss‐of function‐variants and splice‐site variants, as previously described.^1^

## 3 RESULTS

**FIGURE 1:**
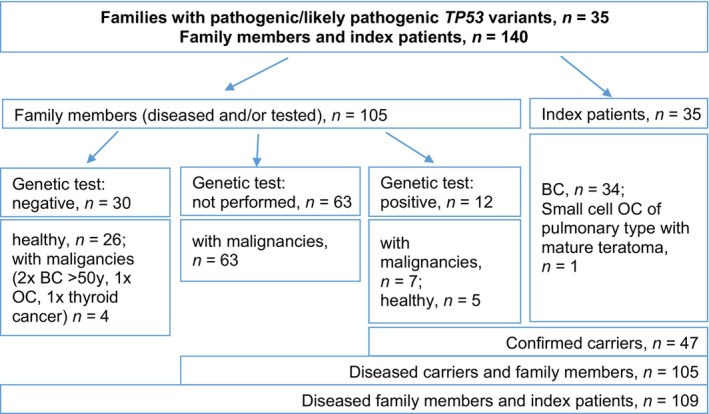
Overview of all families with pathogenic/likely pathogenic *TP53* germline variant from the HBOC Center, Cologne. HBOC = Hereditary Breast and Ovarian Cancer; BC = breast cancer; OC = ovarian cancer; y = years.

The present analysis included 35 index patients with class 4 or 5 *TP53* germline variants and a total of 105 family members from the respective presumptive segregating family branches (Figure 1). All 35 patients and seven of their relatives (*n* = 42) were confirmed as carriers and had an oncological disease. A total of 53 primary BC/DCIS was detected in 38 confirmed *TP53* PV carriers. Table 3A/B shows the clinical characteristics of these BC/DCIS. In total, 149 malignancies were recorded in 105 diseased individuals, including both confirmed carriers and untested family members (Figure 1, Table 4). Of the diseased individuals, 26 had a total of 44 additional malignancies (Table 4). Predictive or targeted testing confirmed the presence of a germline variant by segregation between parents and offspring in 10 out of 35 families (Table 1).

**TABLE 3:** Characteristics and therapy of breast cancer or Ductal carcinoma in situ in *TP53* variant carriers.

(a) BC/DCIS in confirmed *TP53* carriers (*n* = 38) (%)		
Age (median) at first event (Chompret) *n* = 23		32 (22–49) years
Age (median) at first event (GC‐HBOC) *n* = 15		41 (32–56) years
Number	DCIS	9 (17)
	Invasive	44 (83)
	Total	53 (100)
Local recurrence	After BCT	4 (8)
	After mastectomy +/− RTx	0 (0)
Additional BC primaries	Ipsilateral	4 (11)
	Contralateral	10 (26)
Chemotherapy before comprehensive genetic analysis	Yes^a^	20 (80)
	No	3 (9)
	Unknown	2 (8)
cRRM	Yes^b^	7 (18)
	No	24 (61)
	Not applicable^c^	8 (21)

(b) BC/DCIS, all (*n* = 53; invasive *n* = 44, DCIS *n* = 9) (%)			
Stage at presentation		T0	9 (17)
		T1	21 (40)
		T2	16 (30)
		T3/T4/unknown	7 (13)
Histology		NST	35 (66)
		DCIS	9 (17)
		Other	6 (11)
		Unknown	3 (6)
Invasive BC	HER2neu receptor status	Positive	12 (27)
		Negative	23 (52)
		Unknown	8 (18)
	ER/PR	Positive	27 (61)
		Negative	12 (30)
		Unknown	5 (13)
	ER/PR/HER2neu	Negative	6 (14)
	Grading	G1	5 (11)
		G2	21 (48)
		G3	14 (32)
		Unknown	3 (7)
DCIS	Grading	High grade	6 (67)
		Unknown	1 (11)
Surgery of BC/DCIS		BCT	18 (34)
		Mastectomy (+/− rtx)	32 (60)
		Not applicable^d^	3 (6)

Abbreviations: BC, breast cancer; BCT, breast conserving therapy; cRRM, contralateral risk‐reducing mastectomy; Chompret, Chompret criteria fulfilled; DCIS, ductal carcinoma in situ; ER, estrogen receptor; HER2neu, ErB2 receptor; GC‐HBOC, German Consortium for Hereditary Breast and Ovarian Cancer, here: GC‐HBOC criteria fulfilled (Table 4); NST, no special type; PR, progesterone receptor; RTx, radiation therapy.

^a^ 25/35 index patients with chemotherapy and of those, 22 with start 3 weeks to 19 years before blood draw for genetic analysis.

^b^ One case with ipsilateral mastectomy and secondary curatively treated sarcoma of thoracic wall 4 years after BCT and RTx.

^c^ Synchronous bilateral BC (*n* = 2), primarily metastasized BC (*n* = 2), unknown (*n* = 4).

^d^ Incidental finding at cRRM (*n* = 1), primarily metastasized BC (*n* = 3).

**TABLE 4:** Primary malignancies of index patients and diseased family members in the presumptive segregating family branch that fulfilled the Chompret or GC‐HBOC testing criteria.

Primary malignancies	Chompret criteria			GC‐HBOC criteria only			Total malignancies	Total patients (*n* = 105) with at least one of the following malignancies (%)
	Index patients (*n* = 20)	Diseased family members (*n* = 45)	Diseased individuals (*n* = 65)	Index patients (*n* = 15)	Diseased family members (*n* = 25)	Diseased Individuals (*n* = 40)		
	First		Additional	First		Additional		
Breast cancer	19	20	12	15	8	8	82	63 (60)
Brain tumor	0	3	3	0	1	0	7	7 (7)
Sarcoma	0	5	4^a^	0	1	0	10	9 (9)
Gastrointestinal carcinoma	0	6	1	0	3	0	10	10 (10)
Skin cancer	0	2	4	0	2	0	8	7 (7)
Lung cancer	0	0	3	0	2	0	5	5 (5)
Leukemia	0	1	3^b^	0	1	0	5	5 (5)
Lymphoma	0	1	1	0	2	0	4	4 (4)
Other malignancies	1	7	5	0	5	0	18	12 (11)
Total malignanciessss	20	45	36	15	25	8	149	–
Patients with subsequent malignancies (%)	10 (50)	10 (22)	20 (31)	6 (40)	0	6 (15)	–	26 (25)

*Note*: Chompret criteria: Sarcomas, Leiomyomasarcoma of the pelvis, malignant histiocytoma, rhabdomyosarcoma, sarcomas of the femur, thigh, and upper jaw and other soft tissue sarcomas of the lower extremity. Other malignancies, Bladder cancer, cervical cancer, nasopharyngeal carcinoma, ovarian cancer, prostate cancer, renal cell carcinoma, thyroid cancer, unknown. *Note*: GC‐HBOC criteria only: Sarcoma: localization unknown. Other malignancies, renal cell carcinoma, testicular carcinoma, unknown.

Abbreviations: GC‐HBOC, German Consortium for Hereditary Breast and Ovarian Cancer.

^a^ Of those 2times in former radiation field after breast cancer therapy.

^b^ Of those 2times acute myeloid leukemia (AML) after prior chemotherapy.

### 3.1 Familial constellations

The GC‐HBOC criterion of at least two women with unilateral BC, at least one of whom developed the disease before the age of 50 years, was fulfilled in 69% (24/35) of the families, whereas the criterion of at least one woman with BC before the age of 36 years was fulfilled in 57% (20/35) of the families included in the study (Table 2). The adapted Chompret criteria for LFS (Table 1) were met in 57% (20/35) of all families and in 50% (5/10) of those with confirmed parental or offspring segregation (Table 1). In most families with LFS, at least one BC occurred at the age of 30 years or younger (60%; 12/20), of which five families reported additional tumors on the LFS spectrum (criterion a) and seven singular cases (criterion f; Table 1). One of five carriers with de novo variants (i.e., both parents tested negative) fulfilled the adapted Chompret criteria (criterion f). Two families with LFS (criterion b) had affected children with a cancer that would have justified genetic analysis without taking further affected family members into account (rhabdomyosarcoma, 2 years; choroid plexus carcinoma, 3 years; Table 1).

### 3.2 Tumor spectrum

The average follow‐up time from the notification of the genetic analysis results was 18 months for all 42 *TP53* variant carriers with oncological disease. To analyze the number of primary cancers of the diseased individuals from families who did and did not fulfill the Chompret criteria, we included the relatives in the presumptive segregating family branch (Table 4). The median age of onset at first malignancy in the families that fulfilled the Chompret criteria was 32 (19–44) years for index patients and 45 (2–85) years for relatives compared with 41 (32–56) years and 51 (18–95) years, respectively, in families where only the GC‐HBOC criteria were fulfilled. Index patients who fulfilled the Chompret criteria had up to three different malignancies, whereas relatives with and without genetic testing had up to seven different malignancies. We found that 31% (20/65) of the diseased individuals in families that fulfilled the Chompret criteria had at least one additional malignancy, whereas 15% (6/40) of the diseased individuals in families that only fulfilled the GC‐HBOC criteria had multiple malignancies. Almost half of these subsequent malignancies were ipsi‐ or contralateral breast carcinomas (44%; 20/44). No men with BC were observed in the families that participated in this study. Of all the oncologically diseased (probable) variant carriers in the 35 families tested, BC was diagnosed in 60% (63/105) (Table 4). Sarcoma and brain tumor were found in 10% (10/105) and 7% (7/105) of all affected individuals, respectively, and was found more frequently in families that fulfilled the Chompret criteria. Only 4% (4/105) of the patients with an oncological disease were minors (rhabdomyosarcoma, 2 years; choroid plexus carcinoma, 3 years; colon carcinoma, 14 years; melanoma, 16 years), and all four were from families who fulfilled the Chompret criteria.

### 3.3 TP53 carriers with BC or DCIS

In confirmed PV carriers, the first BC/DCIS occurred at a median age of 32 years (22–49 years) if one of the Chompret criteria was fulfilled and 41 (32–56) years if only the GC‐HBOC testing criteria were fulfilled (Table 3A). More than one‐fourth (29%; 11/38) of the patients developed bilateral BC/DCIS and four developed ipsilateral second or tertiary cancers (11%; 4/38). Bilateral BC/DCIS occurred simultaneously in 20% (2/11) of these patients. The percentage of patients with BC and positive hormone receptor status was 61% (27/44), whereas the percentage of those patients with positive HER2neu status was 27% (12/44) in the tested cohort (Table 3B).

### 3.4 Type of TP53 variant

Most of the *TP53* PV (54%, 19/35) belonged to a group of missense variants with no specific category (i.e., other Table S1,S2). The variants in only four families were classified as dominant negative missense variants (11%, 4/35 families), with three of these families fulfilling the Chompret criteria. Age of onset at first malignancy was lower in families with dominant negative (34 [14–60] years) or loss‐of‐function (32 [3–79] years) variants when compared to families with other missense (45 [18–95] years) or low‐penetrance (48 [26–72] years) variants (Table S2).

### 3.5 Predictive and targeted testing

Segregation analysis identified seven family members with malignancies as *TP53* variant carriers, whereas four relatives did not carry the variant (Figure 1). Additionally, a total of 30 predictive genetic tests was performed on oncologically healthy family members, only five of which were found to carry the variant (17%). De novo variants were found in five index cases with negative test results for both parents using predictive testing. Targeted or predictive testing was performed in 21 out of 35 families (66%) and in a total of 42 cases. The mean testing rate was 1.7 per family.

## 4 DISCUSSION

This study provides insights into the family constellations and frequency of malignancies in *TP53*‐positive families who were identified using the GC‐HBOC inclusion criteria for germline testing. It supports earlier reports of *TP53*‐positive families with a less severe phenotype that were identified when the adapted Chompret criteria for LFS were not fulfilled.^15^, ^16^, ^17^

### 4.1 Family constellations

The *TP53* variant carriers identified using the GC‐HBOC criteria mostly were from families with two women who developed unilateral BC, with one who developed the disease before the age of 50 years (69%), and at least one woman who developed unilateral BC before the age of 36 years (57%). These findings were consistent with BC being the most frequent malignancy in female *TP53* variant carriers and a main feature of LFS. Furthermore, early onset of BC before the age of 31 years became a Chompret criterion in 2015 owing to a carrier frequency of 6%.^1^

In our study, 57% of the families fulfilled the LFS criteria according to the Chompret criteria,^1^ whereas only 39% of *TP53* variant carriers were previously reported to meet the Chompret criteria.^16^ Rana et al.^16^ identified *TP53* variant carriers using various gene panel studies to analyze hereditary BC, colon, pancreatic, or renal cancer risk. The group that fulfilled only the criteria for panel testing showed a family history with less tumor disease burden than the comparative group of women who had undergone targeted *TP53* single‐gene testing for suspected LFS.^16^

### 4.2 Age of onset

In our sample of confirmed *TP53* variant carriers from families that fulfilled the Chompret criteria, the median age of the first BC diagnosis was 32 years; it was 41 years in families who fulfilled the GC‐HBOC testing criteria, but not the Chompret criteria. This was consistent with the findings from Rana et al.,^16^ who reported that the median age at onset of first BC was significantly lower in the group after single‐gene testing (33 years) compared to the group of patients with multigene panel testing (40 years). This is also in line with the median age at first onset of BC of variant carriers after targeted testing of women (33 [20–69] years) reported in a large French registry of families with LFS.^1^ Although the confidence intervals overlapped in our study sample, the age of onset of BC in families without LFS is higher, which is indicative of a lower *TP53* penetrance.

The reported median age of onset of first malignancy of females was 28 years after targeted testing and 36 years in the group of panel testing, whereas it was 28 years in the French registry of families with LFS.^1^, ^16^ In our cohort, the median age at first malignancy was slightly higher than indicated in these earlier reports. This could be due to because of the testing criteria and the consecutively low number of children with oncologic disease included in our study. In the French registry, about 40% of the variant carriers with an oncologic disease were younger than 18 years compared with 4% in the present study.^1^

### 4.3 Tumor spectrum

In addition to the analysis of age at first occurrence of disease, examination of the tumor spectrum can be used to investigate the penetrance of a germline variant. Notably, we found that 45% of the recorded additional malignancies were second or third breast carcinomas. The proportion of affected variant carriers with BC was 79% in the study by Bougeard et al.,^1^ which was largely similar to 60% observed in this study. The number of affected individuals with sarcomas (10%) and brain tumors (7%) observed among all affected patients and family members was lower compared to the French registry, where 43% of the patients suffered from sarcomas and 13% from brain tumors.^1^ The lack of other LFS‐typical malignancies could in part be explained by the study sample, including families that met the GC‐HBOC inclusion criteria. It also suggests a lower penetrance of the *TP53* PV in families without LFS.

BC was the most common first and second cancer in female PV carriers both in an earlier study^3^ and in the current study. An analysis of PV carriers from different GC‐HBOC centers, including those from the Center in Cologne, showed a prolonged time to a second BC diagnosis in carriers who had undergone contralateral risk‐reducing mastectomy.^29^ There is considerable overlap of these results with those in the current study, and interpretation justifies the use of larger datasets to develop a better understanding of penetrance in different testing cohorts.^29^

### 4.4 Type of TP53 variant

Clinical and functional data support the impact of *TP53* variant location on disease penetrance.^15^, ^30^ The mean age of tumor onset was lower in carriers with a dominant negative missense mutation (21.3 years) compared to carriers with all types of loss‐of‐function mutations (28.5 years) or genomic rearrangements (25.8 years).^1^ Conversely, low‐penetrance missense mutations such as the Brazilian founder variant have also been reported.^1^, ^31^, ^32^ This variant was present in one family with BC in our study and one other patient who developed BC after the age of 40 years and did not fulfill the Chompret criteria. In our sample, the age of onset at first malignancy was the lowest in families with a loss‐of‐function *TP53* variant (32 years), which was not consistent with earlier reports and might be due to the low number of samples used in our study. The median age of onset of first malignancy in families with dominant negative variants was lower (34 years) than that in families with other missense (45 years) or low‐penetrance (48 years) variants, which was consistent with earlier observations.^1^, ^30^, ^31^, ^32^ As the classification of variants is complex (e.g., dominant negative or loss‐of‐function), the missense variants of the DNA binding domains that are associated with a more severe phenotype of LFS, are defined as “hotspot variants”.^15^ More stringent definitions and knowledge of other modifying factors are urgently needed to guide counseling for disease prevention in patients with de novo germline variants.

The strength of our study is the large amount of pedigree, clinical data, and genetic information that can be applied to the standard of knowledge‐generating care of families with HBOC syndrome in Germany.^18^, ^33^ However, this study also had some limitations. This was a small retrospective study with a partial lack of follow‐up and mostly short follow‐up reporting; therefore, the number of subsequent malignancies may be underestimated. Another limitation was the small proportion of relatives in the presumptive segregating family branch with predictive or targeted testing; part of the malignancies in the family members could be sporadic and the actual age of onset of malignancies in *TP53* variant carriers may be lower.

Predictive or targeted testing was performed in more than half of the families, and five index patients carried de novo variants. However, with a testing rate of 1.7 per family, it was not possible to determine the rate of de novo variants, which are estimated to occur at a frequency of up to 7%–20%.^27^, ^34^ The literature to date has not distinguished between parental mosaic, somatic variants in germ cells that result in a germline variant and de novo mosaic variants in the patients. It is only with the introduction of next‐generation sequencing that some of the previous genetic alterations estimated as germline variants can now be identified as mosaics because of low VFs.^35^ This means that both whole branches of the family as well as part of the patient's body cells would not be affected by the variant, reflecting a lower observed penetrance of the *TP53* variant. The true proportion and kind of de novo variants are difficult to determine because of technical obstacles in testing different tissues and a limited readiness for predictive targeted testing in family members.

Predictive testing identified only 17% of oncologically healthy family members as *TP53* variant carriers. The low penetrance measured using family history and the adapted Chompret criteria could be the result of an unexpectedly high rate of mosaicism. In the present study, only patients with a VF > 30% were included in the analysis. Given that cases of clonal hematopoiesis with a VF > 40% have been reported, the rate of non‐germline variants reported in our study sample might be even higher.^36^ No simple thresholds for determining a mosaic are known; Batalini et al.^25^ suggested a VF‐cutoff at 30% to evaluate the presence of a mosaic, and more recently, a more elaborate effort was published to distinguish non‐germline variants as aberrant clonal expansions from post‐zygotic mosaic.^37^ For practical reasons, Rana et al.^16^ only considered individuals with VFs above 25% without further assessment of the penetrance after gene panel testing, whereas another study group chose a VF of 35% as a cutoff for the analysis of penetrance of prostate cancer in male carriers of a pathogenic *TP53* variant.^38^

The tumor spectrum and efficiency of preventive options for *TP53* PV carriers with suspected lower disease penetrance should be carefully examined in larger prospective cohorts. This work is especially urgent because predictive testing for PVs in *TP53* and cancer surveillance is generally offered to children. A more graded recommendation for predictive testing in children was suggested by the European guideline of ERN‐GENTURIS, which considers the age of onset of malignancies in the family and variant‐specific knowledge about penetrance from the literature.^9^ Owing to the immediate therapeutic consequences and existing prevention options, the extent of somatic cells affected by a *TP53* germline PV is of great clinical importance, especially in patients with early BC.^7^

*TP53*‐associated tumor syndrome is a rare predisposition syndrome, and our analysis provides evidence of a potentially less severe phenotype in families identified by multigene panel testing. Knowledge‐generating care in specialized centers will enable further phenotype analysis and the adaptation of risk‐prevention strategies in the future.

## AUTHOR CONTRIBUTIONS

**K. Kast:** Conceptualization (lead); data curation (lead); formal analysis (lead); investigation (lead); methodology (lead); project administration (lead); resources (equal); validation (equal); writing – original draft (lead); writing – review and editing (lead). **K. Rhiem:** Data curation (supporting); resources (equal); writing – review and editing (supporting). **M. Larsen:** Data curation (supporting); formal analysis (supporting); investigation (supporting); methodology (supporting); resources (supporting). **B. Wappenschmidt:** Data curation (supporting); methodology (supporting); resources (equal); supervision (supporting); validation (supporting); writing – original draft (supporting); writing – review and editing (supporting). **R. Schmutzler:** Funding acquisition (lead); investigation (supporting); methodology (supporting); resources (equal); supervision (supporting); writing – original draft (supporting); writing – review and editing (supporting).

## FUNDING INFORMATION

The GC‐HBOC (German Consortium of Hereditary Breast and Ovarian Cancer) is supported by the German Cancer Aid (grant no 110837 and 70114178, coordinator: Rita K. Schmutzler, Cologne) and the Federal Ministry of Education and Research (BMBF), Germany (grant no 01GY1901). The funding sources had no role in the design and conduct of the study; collection, management, analysis, and interpretation of the data; or preparation, review, or approval of the manuscript.

## CONFLICT OF INTEREST STATEMENT

Karin Kast: Honoraria: MSD; Advisory Board: Pfizer. The other authors have no conflicts of interest to declare.

## ETHICS STATEMENT

All procedures performed in studies involving human participants were in accordance with the ethical standards of the institutional research committee (No. EK 07–048) and with the 1964 Helsinki declaration and its later amendments or comparable ethical standards. This article does not contain any studies with animals performed by any of the authors.

## CONSENT

Informed consent was obtained from all individual participants included in the study.

## Supporting information


Table S1.

Table S2.

## Data Availability

The dataset supporting the conclusions of this article is available upon reasonable request. Requests should be made to Dr. Karin Kast (University Hospital Cologne: karin.kast@uk-koeln.de).
