# Supplementary material for: Phenotype analysis of families with TP53 germline variants at the Center for Familial Breast and Ovarian Cancer, Cologne
Source: Cancer Med. 2024 Jan 17;13(3):e6920. doi: 10.1002/cam4.6920 (PMC10905677; doi:10.1002/cam4.6920)
Supplement: Supplementary file 1 — Table S1. [file CAM4-13-e6920-s001.docx]

**Suppl. Tab 1: *TP53* germline variants (class 4/5) in index patients (n=35) according to the GC-HBOC expert panel for variants of unknown significance (VUS task force)**

| **Nr.** | **cDNA** | **Protein** | **IARC-Class** | **Type of variant** | **Chompret criteria fulfilled** |
| --- | --- | --- | --- | --- | --- |
| **1** | c.560G>C | p.(Gly187Ala) | 4 | MS | no |
| **2** | c.1040C>A | p.(Ala347Asp) | 5 | MS | yes, f |
| **3** | c.473G>A | p.(Arg158His) | 5 | LP | no |
| **4** | c.473G>A | p.(Arg158His) | 5 | LP | no |
| **5** | c.524G>A | p.(Arg175His) | 5 | DN | yes, a |
| **6** | c.542G>A | p.(Arg181His) | 5 | MS | yes, f |
| **7** | c.159G>A | p.(Trp53*) | 5 | LOF NS | yes, f |
| **8** | c.535C>T | p.(His179Tyr) | 5 | DN | yes, a (ad) |
| **10** | c.542G>A | p.(Arg181His) | 5 | MS | no |
| **11** | c.832C>T | p.Pro278Ser) | 5 | MS | no |
| **12** | c.743G>A | p.(Arg248Gln) | 5 | MS | no |
| **14** | c.1024C>T | p.(Arg342*) | 5 | LOF NS | yes, f |
| **15** | c.763_777del | p.(Ile255_Asp259del) | 4 | LOF FS | no |
| **17** | c.1024C>T | p.(Arg342*) | 5 | LOF NS | nein |
| **18** | c.1009C>T | p.(Arg337Cys) | 5 | MS | yes, a (ad) |
| **20** | c.743G>A | p.(Arg248Gln) | 5 | MS | yes., b |
| **21** | c.733G>A | p.(Gly245Ser) | 5 | MS | no |
| **22** | c.455C>G | p.(Pro152Arg) | 4 | MS | yes, a |
| **23** | c.1009C>T | p.(Arg337Cys) | 5 | MS | yes, b (ad) |
| **26** | c.920-1G>A | acceptor Splice site | 4 | LOF SS | yes, b |
| **27** | c.542G>A | p.(Arg181His) | 5 | MS | yes, a |
| **28** | c.542G>A | p.(Arg181His) | 5 | MS | no |
| **31** | c.725G>A | p.(Cys242Tyr) | 5 | MS | no |
| **32** | c.845G>A | p.(Arg282Gln) | 3* | MS | no |
| **33** | c.438G>A | p.(Trp146*) | 5 | MS | yes, a (ad) |
| **34** | c.743G>A | p.(Arg248Gln) | 5 | LOF FS | no |
| **36** | c.1010G>A | p.(Arg337His) | 5 | LP | no |
| **41** | c.242del | p.(Thr81Asnfs*42) | 5 | LOF FS | yes, f |
| **42** | c.493C>T | p.(Gln165*) | 5 | LOF NS | yes, f |
| **43** | c.916C>T | p.(Arg306*) | 5 | LOF NS | yes, b |
| **44** | c.768dupA | p.(Leu257Thrfs*7) | 5 | LOF FS | yes, a (ad) |
| **45** | c.1009C>T | p.(Arg337Cys) | 5 | MS | yes, f |
| **46** | c.1040C>A | p.(Ala347Asp) | 5 | MS | yes, a |
| **47** | c.524G>A | p.(Arg175His) | 5 | DN | yes, a |
| **48** | c.818G>A | p.(Arg273His) | 5 | DN | no |

MS: normal missense variant (Bougeard et al. 2015)

LP: low penetrant effect (Bougeard et al. 2015)

DN: variant with a dominant-negative effect according to the IARC p53 database

LOF: loss-of-function (Bougeard et al. 2015)

NS: nonsense variant

FS: frameshift variant

SS: splice-site variant

a-f: Chompret criteria according to Table 1

ad: adapted according to Bougeard et al. (2015)

GC-HBOC: German Consortium for Hereditary Breast and Ovarian Cancer.

*Variant class 3 with tendency to class 4 (likely pathogenic variant), clinical recommendations in GC-HBOC according to class 4/5 variants.
