# Supplementary material for: Phenotype analysis of families with TP53 germline variants at the Center for Familial Breast and Ovarian Cancer, Cologne
Source: Cancer Med. 2024 Jan 17;13(3):e6920. doi: 10.1002/cam4.6920 (PMC10905677; doi:10.1002/cam4.6920)
Supplement: Supplementary file 2 — Table S2. [file CAM4-13-e6920-s002.docx]

**Suppl. Tab 2: Age of onset of first malignancy in patients that carry different types of *TP53* variants**

| **Type of variant*** | **Number of families** | | | **Number of diseased carriers and family members (n=105) (%)** | **Median age of onset of first tumor (years) in all affected family members (n=105)** |
| --- | --- | --- | --- | --- | --- |
|  | with Chompret criteria (n=20) | with GC-HBOC criteria only (n=15) | total (%)  (n=35) |  |  |
| **Missense variants** | 12 | 14 | 27 (77) | 74 (70) | 45 (14-95) |
| - Other missense variant | 9 | 10 | 19 (54) | 53 (55) | 45 (18-95) |
| - Dominant-negative variants | 3 | 1 | 4 (11) | 10 (10) | 34 (14-60) |
| - Low-penetrance variants | 0 | 3 | 3 (9) | 11 (10) | 48 (26-72) |
| **Loss-of-function variants** | 7 | 1 | 8 (23) | 27 (24) | 32 (3-79) |
| **Splice-site variant** | 1 | 0 | 1 (3) | 4 (4) | 26 (2-72) |

*According to Bougeard et al. (2015).

Abbreviations: GC-HBOC = German Consortium for Hereditary Breast and Ovarian Cancer.
